# WHO Grade 2 Neuroendocrine Tumor in a 15-Year-Old Male: A Case Report and Literature Review

**DOI:** 10.1155/2014/426161

**Published:** 2014-11-30

**Authors:** Eric Johannesen, Van Nguyen

**Affiliations:** Department of Pathology and Anatomical Sciences, University of Missouri, One Hospital Drive, Columbia, MO 65212, USA

## Abstract

Neuroendocrine tumors, distinguished from adenocarcinomas by their neuroendocrine differentiation, are the most common pediatric epithelial malignancy that most often occurs in the appendix. In 2010, the WHO classified neuroendocrine neoplasms into three grades based on morphology, mitotic count, and Ki67 proliferation index. A 15-year-old male with a history of anemia and failure to thrive was diagnosed with a well-differentiated neuroendocrine tumor in the jejunum that invaded into the subserosal soft tissue and metastasized to four lymph nodes. Pediatric neuroendocrine tumors frequently arise within hereditary tumor syndromes with pancreatic neuroendocrine tumors being the most common. Several studies also indicate an elevated risk of small intestinal neuroendocrine tumors in which children born to a parent with a history of neuroendocrine tumors in the small intestine have a significant increased risk of developing one.

## 1. Introduction

Neuroendocrine neoplasms are epithelial tumors with neuroendocrine differentiation. The most current classification separates well-differentiated tumors into G1 and G2 with poorly differentiated malignancies as G3. Although rare, neuroendocrine tumors are the most common pediatric epithelial malignancy. The most common site in the GI tract is the appendix, followed by the hindgut and then the midgut. To date, no case of a jejunal neuroendocrine tumor in a 15-year-old patient has been described in the literature. This paper presents the case of a neuroendocrine tumor in a 15-year-old male, along with a discussion of the classification of these tumors, the areas where they frequently arise, and risk factors for neuroendocrine tumors in pediatric patients.

## 2. Clinical History

A 15-year-old male with a history of failure to thrive presented with anemia and abdominal pain. He initially presented two years ago with a primary complaint of anemia. His family history is significant for carcinoid tumor in his mother, although the grade and location of the tumor were not known. He was initially treated with iron supplementation and was lost to follow-up. Two years later he presented again with anemia, failure to thrive, and abdominal pain. He was referred to gastroenterology. Multiple endoscopies with biopsies were performed with the results ranging from normal to peptic duodenitis and reflux esophagitis. A subsequent capsule endoscopy revealed multiple ulcerations in the mid to distal jejunum. It was then felt that surgical intervention was necessary, so the patient was referred to surgery.

## 3. Operative Findings

On diagnostic laparotomy, a large section of the distal jejunum was shortened with an associated thickened mesentery. Numerous intraluminal and serosal masses were found within the small bowel and mesentery. Multiple, visibly enlarged lymph nodes were present along with areas of small bowel scarring. A small bowel resection was performed along with an appendectomy.

## 4. Pathologic Findings

**Figure 1:**
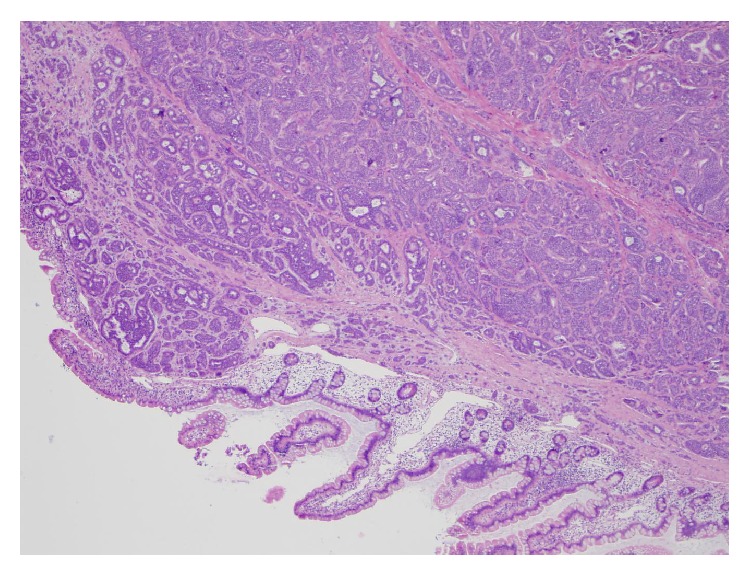
Low power view of neuroendocrine tumor within the submucosa in the jejunum.

**Figure 2:**
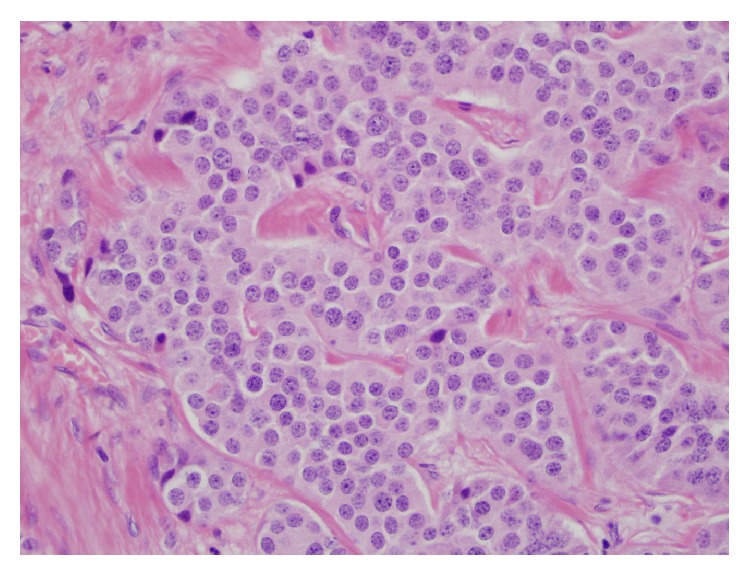
High power view showing cytologically bland cells with round monomorphic nuclei and granular chromatin.

**Figure 3:**
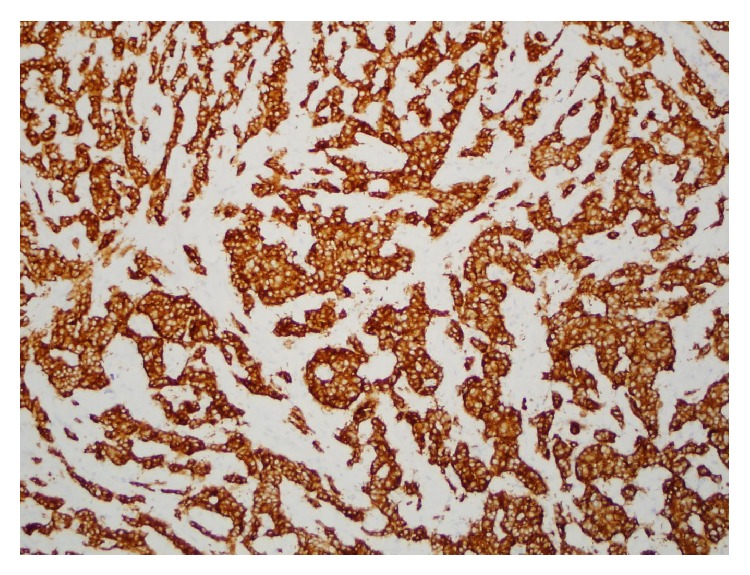
The carcinoma cells are strong and diffusely positive for synaptophysin.

**Figure 4:**
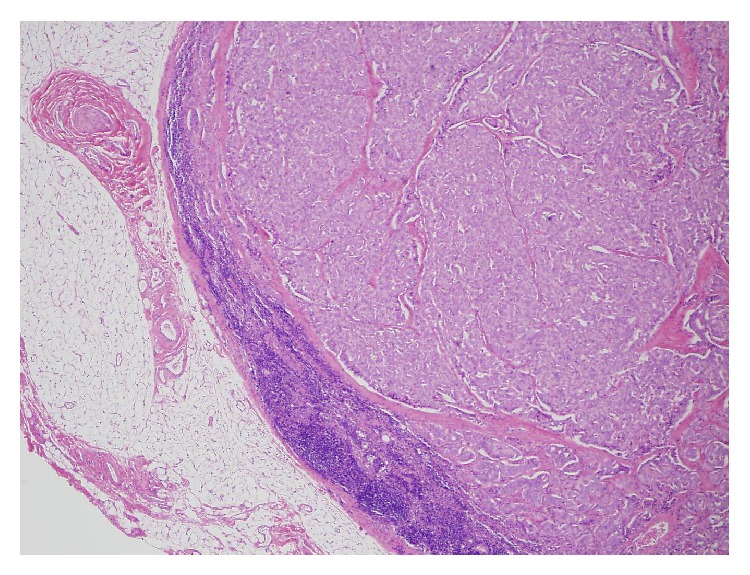
Low power view of metastatic tumor within a lymph node.

The masses seen at laparoscopy had the low power appearance of a well-differentiated carcinoma that was arranged primarily in solid nests. The tumor invaded through the muscularis propria and into the subserosal soft tissue (Figure 1). Multiple foci of tumor, the largest measuring three centimeters, were present in the lamina propria and submucosa as well as at the proximal and distal margins of resection. The tumor was composed of cells with abundant eosinophilic cytoplasm and round nuclei that contained finely stippled chromatin (Figure 2). Focal, mild anisokaryosis was present, but no necrosis was identified. The morphologic features were highly suspicious of a neuroendocrine tumor, so immunoperoxidase studies were performed for confirmation as well as ruling out an adenocarcinoma (Table 1). The tumor was strongly and diffusely positive for synaptophysin (Figure 3) and chromogranin, confirming neuroendocrine differentiation. To determine the grade of the tumor, a mitotic count and a Ki67 immunostain were also performed. The tumor had one mitosis per ten high power fields averaged over 50 high power fields, consistent with a G1. The Ki67 proliferation index was 3.5%, consistent with a G2. Since the proliferation index was of a higher grade, the tumor was graded as a G2. The tumor had metastasized to four lymph nodes (Figure 4), which, along with the tumor size and extent of invasion, was consistent with a more aggressive neoplasm.

## 5. Clinical Follow-Up

Since his surgery in January, he has been doing well. He had good energy and has gained almost nine pounds. An octreoscan performed four months after his operation showed no evidence of tumor recurrence and the most recent chromogranin A level was normal at 70 ng/mL.

## 6. Discussion

Neuroendocrine neoplasms are epithelial tumors with neuroendocrine differentiation, usually confirmed by positivity for neuroendocrine immunohistochemical markers such as synaptophysin and chromogranin.

These tumors used to be grouped into categories based on their morphologic features. The well-differentiated neoplasms were known as carcinoids and the poorly differentiated neoplasms were called large cell neuroendocrine carcinomas and small cell carcinomas.

Recently, it was found that proliferative activity, which included mitotic activity and Ki67 proliferation index, was found to be a useful prognostic indicator that correlated with other features, such as tumor size, invasion, and metastasis [1].

In 2010, the WHO grouped neuroendocrine tumors into three grades based on mitotic activity and proliferation index while maintaining the morphologic distinctions. Well-differentiated neuroendocrine tumors are separated into grade 1 and grade 2 based on mitotic count and/or Ki67 proliferation index. Grade 3 tumors are poorly differentiated large and small cell carcinomas that typically have high mitotic counts and Ki67 proliferation index. These findings are summarized in Table 2 [2]. Although some confusion exists as to how to grade tumors that have a Ki67 index between 2 and 3%, a more recent study validated the Ki67 index criteria for grade 1 and grade 2 neuroendocrine tumors of the WHO classification and found that Ki67 index of 3 percent was the appropriated cutoff separating grade 1 from grade 2 tumors [3]. The case presented here is unusual in that if the grade was determined by mitotic activity alone, this tumor would be a grade 1, but the Ki67 index was 3.2 percent, which would put it at grade 2. Discordant findings like this have occurred in other patients and a study which looked at this found that tumors that were graded as grade 1 by mitotic activity and grade 2 by Ki67 proliferation index frequently had more aggressive morphologic features such as large tumor size and metastasis consistent with grade 2, like in this case [4]. Neuroendocrine tumors most frequently arise in the thyroid, parathyroid, adrenal gland, pancreas, and gastrointestinal tract. Although neuroendocrine tumors are rare in children and adolescents, they are the most common gastrointestinal epithelial malignancy in this group [5]. Among the GI neoplasms, the appendix is the most common site, making up 18% of all neuroendocrine tumors. The hindgut is second with 9% followed by the midgut with 5% [6]. While appendiceal tumors have a low risk of recurrence or metastasis, extra-appendiceal tumors have a higher recurrence and/or metastasis risk [7].

Pediatric neuroendocrine tumors frequently arise in the setting of a hereditary tumor syndrome with up to 30% of cases occurring in this context [8]. Pancreatic neuroendocrine tumors occur as a part of MEN1 syndrome, von Hippel-Lindau syndrome, and neurofibromatosis type 1, while tumors in the stomach have been found in MEN1. Duodenal and ampullary carcinoids have been found in patients with NF1 [8, 9]. Some cases of carcinoid tumors in patients with familial adenomatous polyposis have been reported, but no definitive association has been made [10]. Neuroendocrine tumors of the ileum have also been found to harbor gains in chromosomes 5 and 7, losses at chromosomes 9p and 11q, and mutations in chromosome 18 [11, 12]. Although small intestinal neuroendocrine tumors have not been commonly associated with a tumor syndrome, several studies have indicated that there is a familial risk of small intestinal neuroendocrine tumors. One study found that children of a parent with a neuroendocrine tumor of the small intestine had a tenfold increased risk of having a tumor with a concordant histologic subtype [13]. Another study found that a child of a person with a history of a small intestinal carcinoid tumor has at least four times the risk of developing a neuroendocrine tumor over a child that did not [14]. This may explain why this patient developed a relatively aggressive tumor at a young age considering that his mother had history of a carcinoid tumor.

Neuroendocrine tumors are distinguished from adenocarcinomas by their neuroendocrine differentiation. They are the most common pediatric gastrointestinal epithelial malignancy with the appendix being the most common site. Although small intestinal neuroendocrine tumors are not associated with any of the common hereditary tumor syndromes, it has been found that children with a family history of neuroendocrine tumor in the small intestinal have an increased risk of developing one themselves.

## Figures and Tables

**Table 1:** Differential diagnosis with corresponding immunoprofile.

Tumor type	Cytokeratin	Synaptophysin	Chromogranin
Adenocarcinoma	+	−	−
Neuroendocrine tumor	+	−	−

**Table 2:** 2010 WHO classification of neuroendocrine neoplasms [2].

Grade	Mitotic count (per 10 high power fields)	Ki67 proliferation index
G1	Less than 2	2% or less
G2	2–20	3–20%
G3	Greater than 20	Greater than 20%
